# Fatigue-induced Fos immunoreactivity within the lumbar cord and amygdala decreases after С_60_ fullerene pretreatment

**DOI:** 10.1038/s41598-020-67034-1

**Published:** 2020-06-17

**Authors:** Andriy V. Maznychenko, Nataliya V. Bulgakova, Inna V. Sokolowska, Kamila Butowska, Agnieszka Borowik, Olena P. Mankivska, Jacek Piosik, Tomasz Tomiak, Olga O. Gonchar, Volodymyr O. Maisky, Alexander I. Kostyukov

**Affiliations:** 1 Department of Movement Physiology, Bogomoletz Institute of Physiology, Bogomoletz Str. 4, Kyiv, 01024 Ukraine; 2 Department of Physical Education, Gdansk University of Physical Education and Sport, Kazimierza Gorskiego Str. 1, Gdansk, 80-336 Poland; 3 Laboratory of Biophysics, Intercollegiate Faculty of Biotechnology UG-MUG, Abrahama 58, Gdansk, 80-307 Poland; 4 Department of Cytology, Bogomoletz Institute of Physiology, Bogomoletz Str. 4, Kyiv, 01024 Ukraine; 5 Department of Hypoxic States Investigation, Bogomoletz Institute of Physiology, Bogomoletz Str. 4, Kyiv, 01024 Ukraine

**Keywords:** Biotechnology, Neuroscience, Physiology

## Abstract

The fundamental aspects related to the mechanisms of action of C_60_ fullerene nanoparticles on the level of the central nervous system in different experimental conditions are still unclear. Electrophysiological investigation and immunohistochemical techniques of *c-fos* expression were combined to determine which neural elements within the lumbar segments and in the central nucleus of the amygdala (CeA) are activated under skeletal muscle fatigue development with prior application of C_60_ fullerenes (dissolved in dimethyl sulfoxide and in distilled water, FDS). After high-frequency electrical stimulation of the triceps surae muscle, the main fatigue-related increases in the c-Fos expression level were registered ipsilaterally within lamina 1 and 5 of the lumbar segments and within the contralateral capsular part of the CeA. C_60_ fullerene pretreatment in animals with subsequent electrical stimulation induced a distinct (2–4 times) decrease in the level of Fos immunoreactivity in the observed structures in comparison with only fatigue-induced rats. It can be supposed that FDS, as antioxidant compound, can decrease the concentration of free radicals in fatigued tissue and reduce the transmission intensity of nociceptive information from muscles to the spinal cord and amygdala, thereby changing the level of c-Fos expression within the lumbar segments and CeA.

## Introduction

Since the discovery of C_60_ fullerene nanoparticles and it free-radical scavenger function was revealed, scientists have been searching for biomedical applications of compounds^1–3^. Thus, the photochemical properties of C_60_ have been revealed as a photosensitizer in anti-cancer photodynamic therapy^3^ and even dermatological and cosmetic applications^4^. Because C_60_ fullerenes have unique properties, a large variety of biological applications have been considered; however, the fundamental aspects related to the mechanisms of action of C_60_ fullerenes on the level of the central nervous system (CNS) in different experimental conditions are still unclear. Recently, we studied the effects of C_60_ fullerenes on exercise tolerance and contractile property changes in rat triceps surae (TS) muscles during the development of muscle fatigue^5,6^. In the electrophysiological and biochemical studies, we have shown that application of the C_60_ fullerenes led to a reduction in the recovery time of the muscle contraction force and an increase in the duration of the muscle endurance under fatigue development. Based on the data obtained from these experiments, we hypothesized that the action of C_60_ fullerenes can affect the central nociceptive processes initiated by skeletal muscle fatigue and induce changes in the neuronal activity patterns in the spinal cord and the brain. The post-fatiguing changes in the CNS are presumably initiated predominantly by fatigue-induced inflow of the nociceptive signals from the high-threshold muscle afferents^7^.

One of the brain structures that is associated with not only emotional information processing but also the processing and modulation of pain sensation is the amygdala. The central nucleus of the amygdala (CeA) receives nociceptive, mechanical and thermal information via the spino-parabrachio-amygdaloid pain pathway and a major output nucleus of the amygdala^8,9^. That is, highly processed information generated in the lateral and basolateral amygdala is transmitted to the CeA, which performs major amygdala output functions and projects signals to pain modulatory systems through forebrain and brainstem connections^10^. Furthermore, the laterocapsular division of the CeA (also known as the “nociceptive amygdala”) receives nociceptive-specific information from the spinal cord and brainstem^11^.

The expression of immediate early genes, especially *c-fos*, is widely used for mapping functional pathways, including the circuitry that underlies the transmission of nociceptive information in the CNS. The proto-oncogene *c-fos* is expressed in neurons in response to various stimuli, and its c-Fos-protein product can be easily detected with immunohistochemical techniques^12–14^. Therefore, studies have combined the electrophysiological investigative and immunohistochemical methods of *c-fos* expression (as a marker of neuronal activation) to determine which neural elements are activated.

It is known that in biological studies, dimethyl sulfoxide (DMSO) is widely used as a solvent and does not show high toxicity at low concentrations^15,16^. In our study, C_60_ fullerenes were dissolved in DMSO and then in distilled water. Thus, the aim of the present study was to reveal the effect of C_60_ fullerene DMSO solution (FDS) on changes in the level of neuronal activity within the L4 and L5 lumbar spinal segments (the main regions of TS afferent projection) and CeA initiated by afferent nociceptive signals from long-lasting contractions of the TS muscles during fatigue development.

## Methods

### Sample preparation, UV-visible spectrophotometry and atomic force microscopy visualization

The appropriate amount of C_60_ fullerene (purity >99.99%, Sigma-Aldrich, Germany) was suspended in DMSO (purity >99.99%, Sigma-Aldrich, Germany) in glass test-tubes to determine its maximum solubility in a tested organic solvent. Four different concentrations were analyzed: 1 mg/mL, 1.5 mg/mL, 1.75 mg/mL and 2 mg/mL. Afterwards, prepared samples were treated with an ultrasonic bath (Polsonic, Warsaw, Poland) for 35–45 min at a power of 620 W and a frequency of 50 Hz to obtain a visibly brown solution. Increases in the ambient temperature were controlled by the addition of ice. It was noticeable that only a concentration of C_60_ fullerene equal to 1 mg/mL was dissolved without any impurities, appearing after sample centrifugation; therefore, this concentration was chosen for further analysis. The final stock solution was stored in the dark at 4 °C.

To characterize the composition and stability of the C_60_ fullerene dissolved in DMSO, repeated spectrophotometric experiments were performed. The absorption spectra of the C_60_ fullerene solution were assessed by a broad range of concentrations (final concentration 0.078 mg/mL) in 0.2 M sodium-phosphate buffer (pH = 6.8). Additionally, the absorption spectra recorded over several days overlapped, which suggests that the C_60_ fullerene DMSO solution remained stable. It was also revealed that the DMSO solution alone did not absorb light in the tested wavelength range.

**Figure 1:**
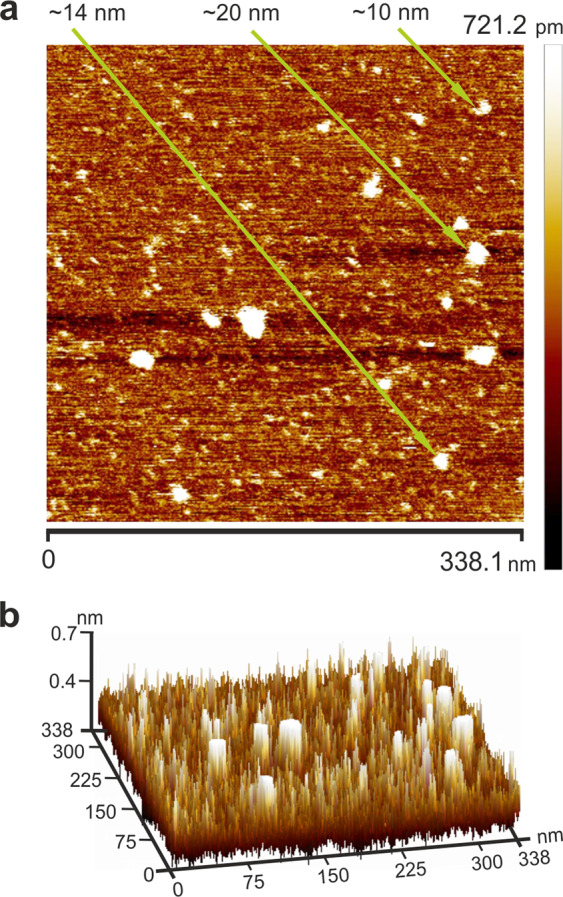
The atomic force microscopy images of C_60_ fullerene nanoparticles on a mica surface precipitated from 1 mg/mL DMSO solution (**a**). The atomic force microscopy images of C_60_ fullerene nanoparticles on a mica surface precipitated from 1 mg/mL DMSO solution, in a three-dimensional view (**b**).

The precise dimensions of the C_60_ fullerene nanoparticles dissolved in DMSO were controlled by atomic force microscopy (Fig. 1a,b). C_60_ fullerene aggregates were deposited onto a freshly cleaved mica substrate by precipitation from a droplet of DMSO solution. Measurements were conducted in the ScanAsyst mode in air (SCANASYST-AIR probes, spring const. 0.4 N/m) using a Bruker BioScope Resolve microscope after complete evaporation of the liquid.

### Procedure and experimental groups

Male Wistar rats weighing 290–340 g were used in the study. The animals were purchased from a state-controlled animal farm through the common animal facility of Bogomoletz Institute of Physiology (Kyiv), were housed in Plexiglas cages and kept in an air-filtered and temperature-controlled (21 ± 1 °C) room under 12 h light/12 h dark conditions. The rats received a standard pellet diet and water *ad libitum*. The use of the animals was approved by the Biomedical Ethics Committee of the Bogomoletz Institute of Physiology and performed in accordance with the European Communities Council Directive of 24 November 1986 (86/609/EEC).

**Figure 2:**
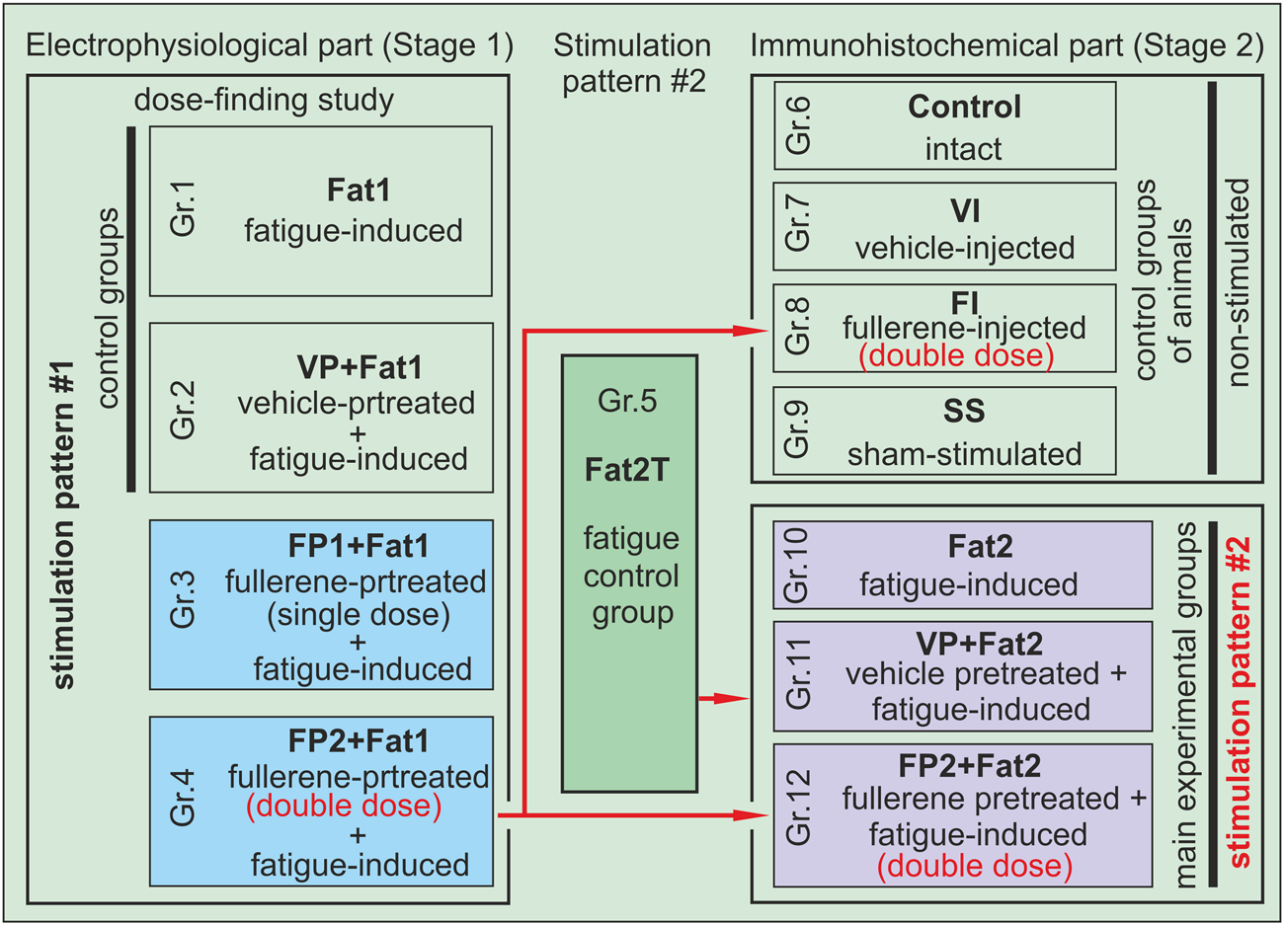
Schematic representation of the study. The dose determined for animals of group 4 was used for groups 8 and 12. Designations: Gr.1–Gr.12 – groups of rats (Fat1 – fatigue-induced; VP + Fat1 – vehicle-pretreated and fatigue-induced; FP1 + Fat1 – fullerene-pretreated (single dose) and fatigue-induced; FP2 + Fat1 – fullerene-pretreated (double dose) and fatigue-induced rats; Fat2T – fatigue-tested; control – intact animals; VI – vehicle-injected; FI –fullerene-injected (double dose); SS – sham-stimulated; Fat2 – fatigue-induced animals; VP + Fat2 – vehicle-pretreated and fatigue-induced; FP2 + Fat2 – fullerene-pretreated (double dose) and fatigue-induced). Fat1 and Fat2 mean that the animals were stimulated using stimulation pattern 1 or 2, respectively. FP1 and FP2 mean that the rats were injected with single or double dose fullerene, respectively.

The work was carried out in 2 stages (Fig. 2). In the first stage, it was necessary to determine the dose of FDS, which can have a significant impact on the development of TS muscle fatigue. For this purpose, stimulation pattern 1 was used (5 stimulation series lasting 30 min each). In the second stage, it was necessary to reveal neural activation (in the studied CNS structures) induced by the electrical stimulation of the TS muscles in the conditions of FDS action. To minimize the proprioceptive and nociceptive influences on c-Fos protein expression in the spinal cord and brain, a stimulation pattern with a relatively short duration, stimulation pattern 2 (30 min of high-frequency stimulation), was used in comparison with stimulation pattern 1, and no surgical intervention was performed.

All animals were randomly divided into 12 groups (1–4 groups – fullerene dose-finding rats; 5–12 groups – animals for immunohistochemistry (IHC): group 1 – fatigue-induced (Fat1) animals (muscle fatigue induced by electrical stimulation, stimulation pattern 1), n = 6; group 2 – vehicle-pretreated and fatigue-induced (VP + Fat1) rats (the animals were i.p. injected with DMSO dissolved in distilled water 1 h prior to electrical stimulation), n = 6; group 3 – fullerene-pretreated and fatigue-induced (FP1 + Fat1) rats (the animals were i.p. injected with 0.15 mg/kg C_60_ fullerenes in DMSO solution dissolved in distilled water 1 h prior to electrical stimulation); group 4– fullerene-pretreated and fatigue-induced (FP2 + Fat1) rats (the animals were i.p. injected with 0.3 mg/kg C_60_ fullerenes in DMSO solution dissolved in distilled water 1 h prior to electrical stimulation); group 5 – fatigue-tested (Fat2T) animals (fatigue-induces stimulation for IHC, stimulation pattern #2), n = 6; group 6 – control (intact) animals for IHC, n = 6; group 7 – vehicle-injected (VI) rats (animals with i.p. injection of DMSO solution dissolved in distilled water), n = 6; group 8 – C_60_ fullerene-injected (FI) rats (animals with i.p. injection of 0.3 mg/kg C_60_ fullerenes in DMSO solution dissolved in distilled water), n = 6; 9^th^ – sham-stimulated (SS) animals (rats with wire electrodes inserted into the TS muscles, but no electrical stimulation was applied), n = 6; 10^th^ – fatigue-induced animals (Fat2), n = 6; 11^th^ – vehicle i.p. pretreated and fatigue-induced (VP + Fat2) rats, n = 6; and 12^th^ – fullerene (0.3 mg/kg) i.p. pretreated and fatigue-induced (FP2 + Fat2) rats. Note that in all cases, the final DMSO concentration in the injected solutions was 5%.

### Operation and electrical stimulation protocols

Stage 1. The animals in groups 1–4 were anaesthetized with ketamine (100 mg/kg “Pfizer”, USA) combined with xylazine (10 mg/kg, “Interchemie”, Holland). The left TS muscles were separated from the surrounding tissue, and their tendons were detached at the distal insertions. The *n. tibialis* was separated from the tissue and cut proximally, and all branches of the nerve, except those innervating the TS, were cut^6^. Thereafter, the rats were gently fixed to the platform. Then, the nerve was mounted on a bipolar platinum wire electrode for electrical stimulation. The hindlimb muscles and nerves were covered with paraffin oil in a pool formed from skin flaps. The TS muscles were connected via the Achilles tendon to the servo-control muscle puller. The muscle tension was measured by semiconductor strain gauge resistors glued to a stiff steel beam mounted on the moving part of a linear motor^6^.

To induce muscle fatigue in rats of these groups (stimulation pattern 1), 5 intermittent high-frequency electrical stimulation series (each with a 30 min duration, separated by rest intervals of 10 min) were used^6^. Each series consisted of trains of 0.2-ms rectangular pulses at a rate of 40 s^−1^ with a duration of 12.4 s that were separated by 5 s intervals of rest. The stimulus current was set to 1.3–1.4 times the motor threshold. After the 12.4-s stimulation period, the muscle was stretched, and the change in length had a bell-shaped form (one period of a 4 Hz sinusoidal signal with corresponding phase locking) with an amplitude of 3.5 mm and a duration of 2 s. The muscle reaction to the stretches appeared as a tension increase after continuous stimulation. These stretches were applied before the poststimulation twitches to remove, or at least diminish, the after-effects remaining from the continuous stimulation^17^. The signals (stimulus pulses, muscle tension and other signals) were sampled via a DAC-ADC device (CED Power 1401). Data acquisition was performed using the program “Spike2” (CED). Input signals were digitized at rates of 5 kHz (muscle tension) and 1 kHz (other signals). Data analysis, including statistical analysis and graph plotting, was performed using the program Origin 8.0 (Origin Lab Corp., USA).

Stage 2. The rats in groups 5 and 9–12 were anaesthetized in the same manner as the animals in groups 1–4 were. To determine the muscle tension under fatigue development (only in the rats in group 5), the TS muscles were isolated from adjacent tissue and connected by a steel thread to a force transducer for recording the isometric contraction forces (as for rats in groups 1–4). In addition (for groups 5 and 9–12), two stimulating electrodes consisting of lacquered silver wires with a diameter of 0.15 mm were placed into a thin hypodermic injection needle, the insulation coating was removed over a length of 2 mm from the ends, and the wires were bent. The needles were inserted into the TS muscles and then withdrawn. The wires remained fixed in the muscles by their hook-like ends (the interelectrode distance was approximately 5 mm). To minimize the motion during fatiguing stimulation, the animals were fixed to the platform. Stimulation of the TS muscles (stimulation pattern #2)^18^ was performed for rats in groups 5 and 10–12 by trains of 0.2 ms rectangular pulses at a rate of 100 s^−1^. Thirty stimulation sessions with 40 s periods of intermittent stimulation and 20 s rest periods were applied. The stimulus current was set to 1.3–1.5 times the motor threshold, which was lower than the threshold for the electrical activation of group III and IV afferents in the somatic nerves of rats^19^. This mode of stimulation was followed by a remarkable decrease in muscle tension.

### Fos immunohistochemistry

At 1.5 h after the substance injections and/or the end of the electrical stimulation period, the animals in groups 6–12 were deeply anaesthetized with sodium pentobarbital (75 mg/kg, i.p., Nembutal, USA) and perfused through the ascending aorta with 0.9% physiological saline, followed by fixative solution containing 4% paraformaldehyde in 0.1 M phosphate buffer (PB) (pH 7.4). Blocks of the lumbar spinal cord and brain were quickly removed, postfixed overnight in the same fixative and cryoprotected in phosphate-buffered sucrose at 4 °C for 48 h. Brain and lumbar segments were cut into coronal frozen sections that were 40 µm thick and collected in wells containing cold phosphate-buffered saline (PBS) (0.1 M of PB containing 0.9% NaCl, pH 7.4) for subsequent immunohistochemical processing. Fos-immunoreactive (Fos-ir) spinal neurons were detected according to a standard avidin-biotin-peroxidase technique^20^ using a rabbit polyclonal antibody directed against the c-Fos protein (1:2000, Ab-5, PC38, Oncogene Research, USA) and a commercial kit (ABC Kit, PK 4001, Vector Laboratories, USA). Briefly, following several rinses in PBS, the sections were placed in PBS containing 3% normal goat serum (Sigma, USA) and 0.3% Triton X-100 (Sigma, USA) for 30 min at room temperature. Free-floating sections were subsequently incubated for 48 h at 4 °C in primary antiserum directed against the c-Fos protein diluted (1:2000) in PBS containing 3% normal goat serum and 0.4% Triton X-100. The sections were then incubated in biotinylated goat anti-rabbit immunoglobulin G (1:200) and avidin-biotin complex (ABC) using a standard protocol. Fos-immunoreactive nuclei were visualized with nickel-intensified 3,3′- diamino-benzidine tetrahydrochloride (DAB, Sigma, USA). The sections were incubated in 0.05 M Tris-buffer (pH 7.4) containing 0.035% DAB, 0.2% nickel ammonium sulfate and 0.005% hydrogen peroxide for 10 min at room temperature to produce a purple-black reaction product, washed in PBS, and mounted into gelatin-subbed slides, air-dried, cleared in xylene and finally coverslipped.

### Statistics

In the electrophysiological study (rats in groups 1–4), each stimulation series was averaged (100 stimulations in one series). The average value of the first series was set to 100%, and the other series were normalized in relation to this value. In a previous study^6^, it was found that until the moment of a drastic decrease in *TS* contraction force, some animals maintained a certain level of muscular contraction force throughout 5–6 series of electrical stimulation. Therefore, for further analysis, data from the first 5 series were selected. Mean values (mean ± s.d.) of the *TS* muscle strength after FDS and DMSO solution induction and those for the condition without any induction were compared using two-way statistical analysis of variance (ANOVA). A Bonferroni *post hoc* analysis was used to determine the differences between groups. The level of significance was set at P < 0.05.

To estimate the effect of electrical stimulation pattern 2 (animals in group 5) on *TS* muscle fatigue development, one-way ANOVA and Bonferroni *post hoc* analysis were used to compare the obtained values (mean ± s.d.).

In the immunohistochemical study (rats in groups 6–12), Fos-ir labeled neurons in the layers of the spinal gray matter and in the central nucleus of amygdala were detected as black nuclei and calculated using an optic microscope at magnifications of × 250 and ×400; their localization was identified according to the atlas^21^. Up to 10 sections from L4 and L5 segments and 5 sections from amygdala at the separate levels from the bregma^21^ per rat of 6–12 groups were taken for analysis. The mean number ± s.e.m. of Fos-ir neurons per section was calculated in layers 1–10 of the spinal gray matter on both sides of the lumbar (L4 and L5) segments and in the central nucleus of the amygdala on different levels. The potential double-count errors of the same cell in the neighboring section were corrected using the Abercrombie equation^22^. The data were analyzed with three-way ANOVA. The factors of variation included three conditions: the groups of animals, spinal or brain segments and ipsi/contralateral sides. Newman-Keuls *post hoc* analysis was used when a significant difference was detected. Values with P < 0.05 were considered to be significant.

## Results

### Electrophysiological study

**Figure 3:**
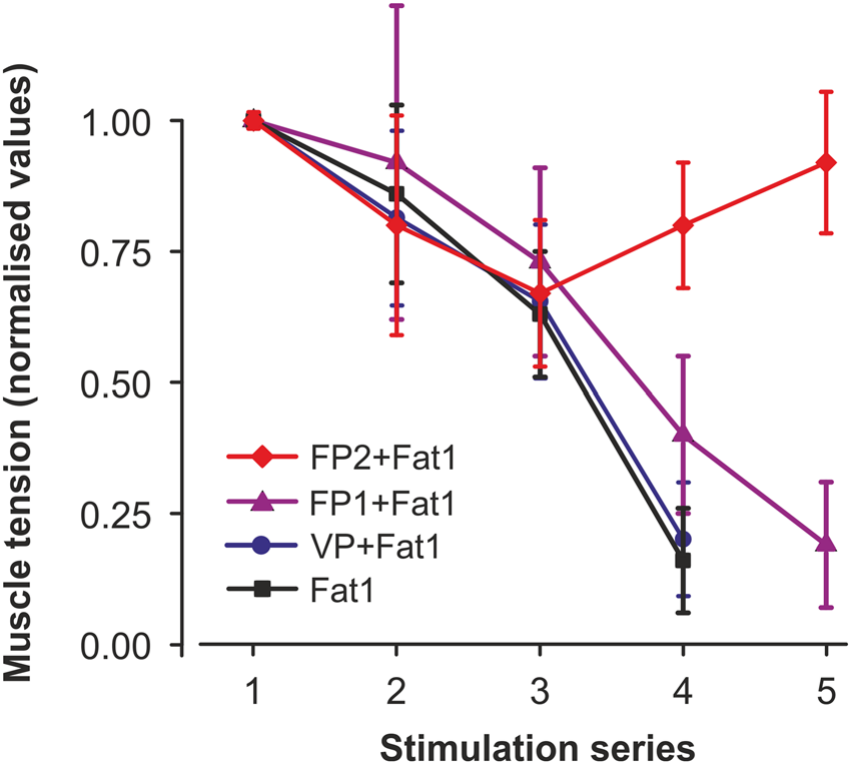
Averages (mean ± s.d.) of the normalized values (with respect to the average values in the first stimulation series) of the TS muscle strength obtained from the animals in the Fat1, VP + Fat1, FP1 + Fat1 and FP2 + Fat1 groups. The values of six animals in each group were averaged by stimulation series. 1–5 – series of the fatiguing electrical stimulations.

**Figure 4:**
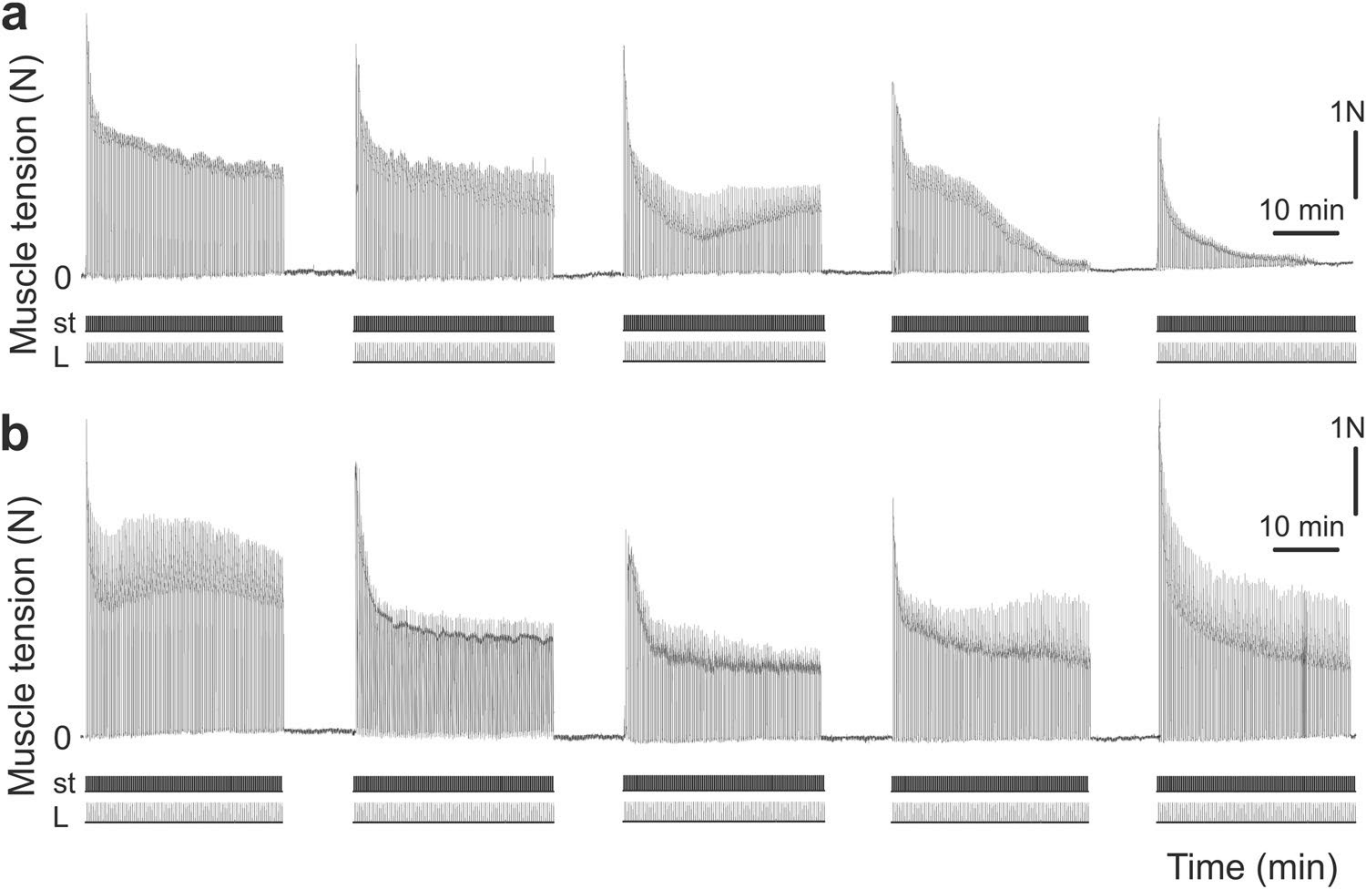
Strength of the muscle contractions after preliminary administration of 0.15 mg/kg (**a**) and 0.3 mg/kg (**b**) FDS. N – muscle force (Newton), st – stimulation mark, L – muscle length (stretching).

Stage 1. During the electrophysiological studies, it was found that in animals of the Fat1 and VP + Fat1 groups, during 4 series (4 × 30 min) of intermittent high-frequency electrical stimulation (stimulation pattern #1), the contraction force of the TS muscles decreased until the muscles ceased to show signs of contraction (Fig. 3). In the first series of stimulations, a gradual decrease in the level of TS muscle activity was observed within 30 minutes. In the second series of stimulations, the amplitudes of the tetanic contractions were somewhat restored; however, the amplitudes did not reach the initial amplitude and continued to decline. In the third series of stimulations, a sharp decrease in muscle strength was observed. During this time period the significant decrease in the level of strength was observed with respect to the first series of stimulations, P < 0.05 (Fig. 3). In the fourth series of stimulations, the isometric contraction of muscle strength continued to decline without further recovery. Statistically significant differences in the strength of the muscle contractions between the rats in groups 1 and 2 were not found, P > 0.05. However, preliminary administration of 0.15 mg/kg FDS in the rats in the FP1 + Fat1 group increased the duration of active work of the muscles for 30 min until it completely reduced the contraction force (Fig. 4a) compared with those of the animals in groups 1 and 2. With the preliminary injection of 0.3 mg/kg FDS (FP2 + Fat1 group), the TS muscles maintained the same constant force level during the five series of fatigue stimulations (Figs. 3 and 4b). Note that statistically significant differences in the strength of muscle contractions between the rats in groups 4 and 1–3 were found after 4 and 5 series of electrical stimulations, P < 0.05. Considering the fact that the injection with a dose of 0.3 mg/kg FDS had the largest influence on the duration of muscle contractions, this dosage was used for further IHC studies.

**Figure 5:**
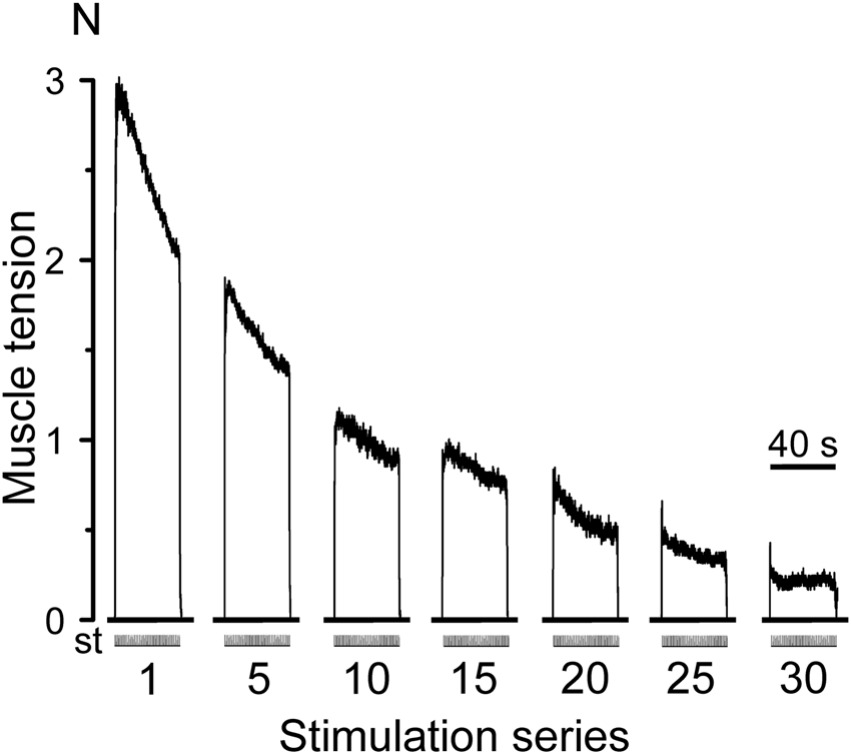
Tension dynamics of the triceps surae muscles during isometric contractions induced by intermittent 30-min electrical stimulations. N – muscle force (Newton); st – stimulation mark.

Stage 2. For the IHC study, electrical stimulation pattern 2 was used. Row traces of TS muscle tension during 30 min of intermittent intramuscular stimulation are shown in Fig. 5. The muscle tension declined within each individual 40-s stimulation session. Compared with the first stimulation session, the 30th session showed a decline in peak tension by 85%.

### c-Fos immunoreactivity in the lumbar segments

**Figure 6:**
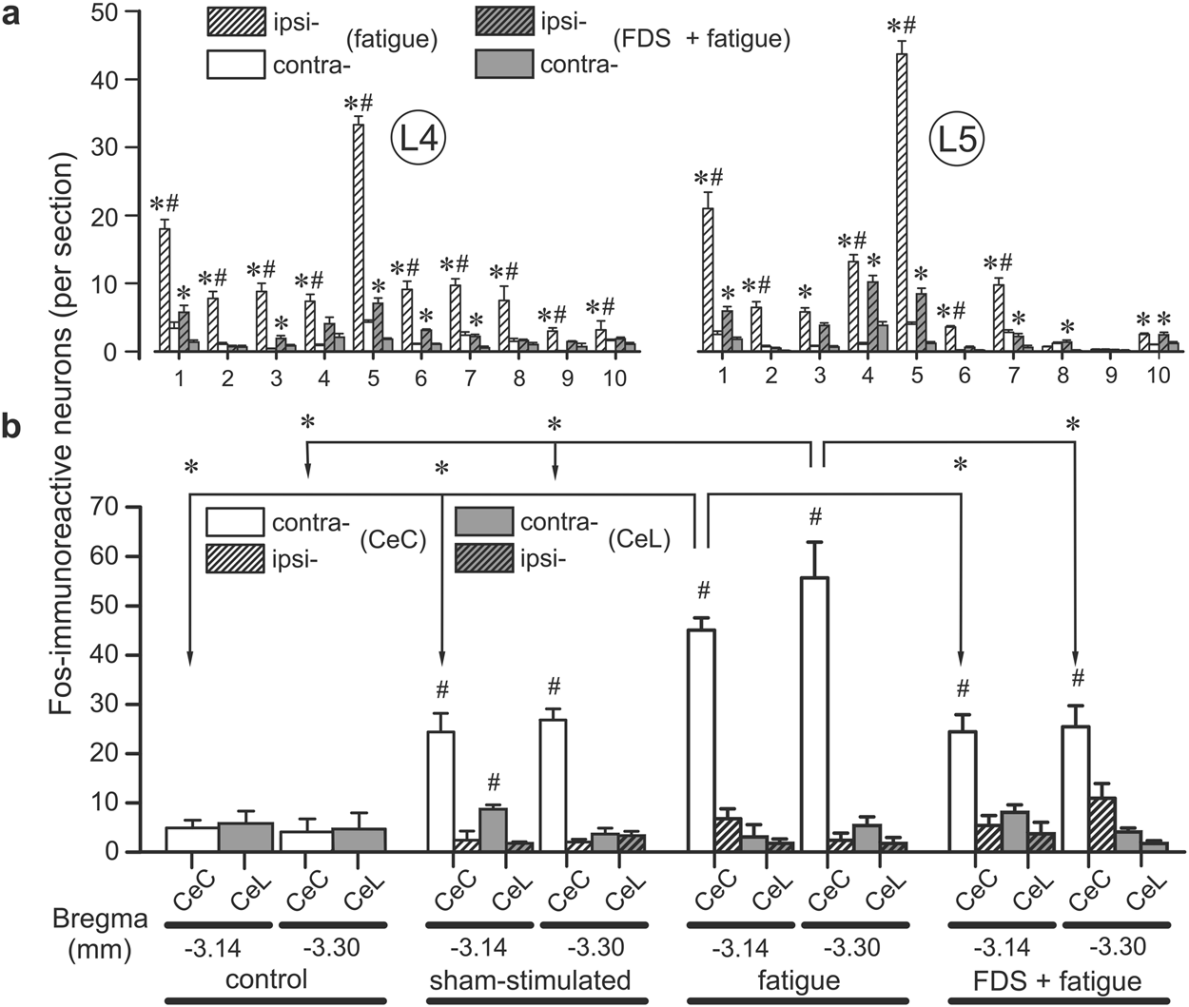
Mean number ± s.e.m. of Fos-ir neurons in the lumbar (L4 and L5) segments and within the capsular (CeC) and lateral (CeL) subnuclei of the central amygdaloid nucleus. The mean number of labeled neurons in the ipsilateral and contralateral sides of L4 and L5 after fatiguing stimulation (Fat2 group, white columns) and after the FDS injection with subsequent fatiguing stimulation (FP2 + Fat2 group, gray columns) is shown in (**a**). The mean number of Fos-ir cells in the CeC (white columns) and CeL (gray columns) for the Control, SS, Fat2 and FP2 + Fat2 animal groups is shown in (**b**). Striped and non-striped columns denote the ipsilateral and contralateral sides of the spinal cord and brain. For the control animals, data are presented unilaterally. The asterisks (*) that are placed on the columns and arrows indicate a significant difference in the number of labeled cells between the different animal groups in the same lamina or structure, P < 0.05. The # symbol represents the difference in the number of positive neurons between the ipsilateral and contralateral sides of the spinal cord and the brain. 1–10 – laminae of the spinal gray matter. −3.14 and −3.30 mm – levels from bregma^21^.

**Figure 7:**
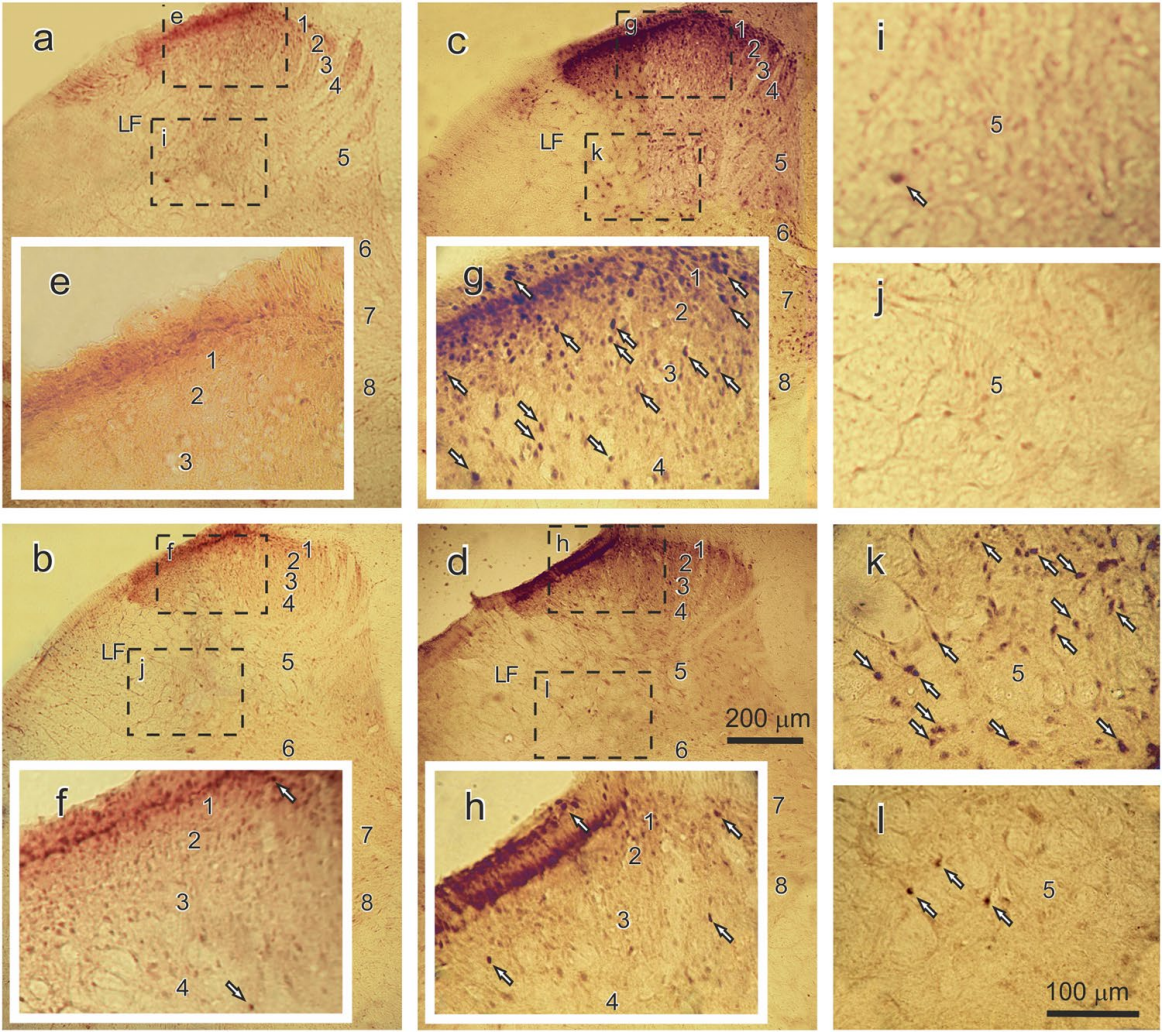
Photomicrographs of the frontal sections of the L4 lumbar segments of the spinal cord (ipsilateral side) in control (**a**), in sham-stimulated rat (**b**) and in animal after electrical stimulation (**c**), and after FDS injection with subsequent fatiguing stimulation (**d**). The rectangular regions enclosed by dashed lines in (**a–d)** are shown in (**e–l**) at higher magnification. Examples of the labeled nuclei of the Fos-immunoreactive (Fos-ir) neurons are shown by arrows. Structures: 1–8 – laminae of gray matter; LF – lateral funiculus. Scale bars: 200 μm in (**d**) also appreciated for (**a–c**) and 100 μm in (**l**) appreciated for (**e–k**).

In the control rats (group 6), the basal level of c-Fos expression on both sides of the L4 and L5 spinal segments was very low (approximately 4–8 Fos-ir neurons per 40-µm-thick section). In contrast, in the animals in the VI, FI and SS groups, the mean number of Fos-ir neurons (per section), as a response to injection-related pain, was some higher than that of the control rats. Activated neurons were detected on both the ipsilateral and the contralateral sides (6.3 ± 0.8 and 3.7 ± 0.9 cells, respectively). Statistically significant differences in the mean number of activated neurons within lumbar segments of the rats in these groups were not found, P > 0.05 (Figs. 6a and 7a,b,e,f,i,j). In contrast, the TS muscle electrical stimulations induced a pronounced increase in the mean number of Fos-ir cells within the studied lumbar segments of the Fat2 and VP + Fat2 groups of rats compared with those of the rats in groups 6–9. Many positive neurons were observed in all laminae of the L4 and L5 segments. However, the highest number of Fos-ir cells was found in the marginal zone and neck of the dorsal horn (laminae 1 and 5, Figs. 6a and 7c,g,k). The mean number of Fos-ir neurons in ipsilateral laminae 1 and 5 of the L4 and L5 segments reached 18.1 ± 1.4 and 33.3 ± 1.3 cells within the L4 segment, respectively, and 21.2 ± 2.4 and 43.7 ± 1.9 within the L5 segment, respectively. Moreover, labeled Fos-ir nuclei (3.2 ± 1.3 units) were also observed in the motoneuronal pools of the L4 segment (Fig. 6a). Note that statistically significant differences in the mean number of Fos-ir neurons between the rats in the Fat2 and VP + Fat2 groups were not found, P > 0.05. The intraperitoneal pretreatment in the animals of the FP2 + Fat2 group induced a statistically significant ipsilateral decrease in the mean number of Fos-ir neurons in the L4 segment within laminae 1–10 and in the L5 segment within laminae 1, 2, 4–8 and 10 compared with those of the rats in the Fat2 and VP + Fat2 groups. For example, the mean number of activated cells was 5.8 ± 0.9 and 7.1 ± 0.7 in laminae 1 and 5 of the L4 segment, respectively, and 5.9 ± 0.7 and 8.5 ± 0.8 in laminae 1 and 5 of the L5 segment, respectively (Figs. 6a and 7d,h,l).

### c-Fos immunoreactivity in the central nucleus of the amygdala

**Figure 8:**
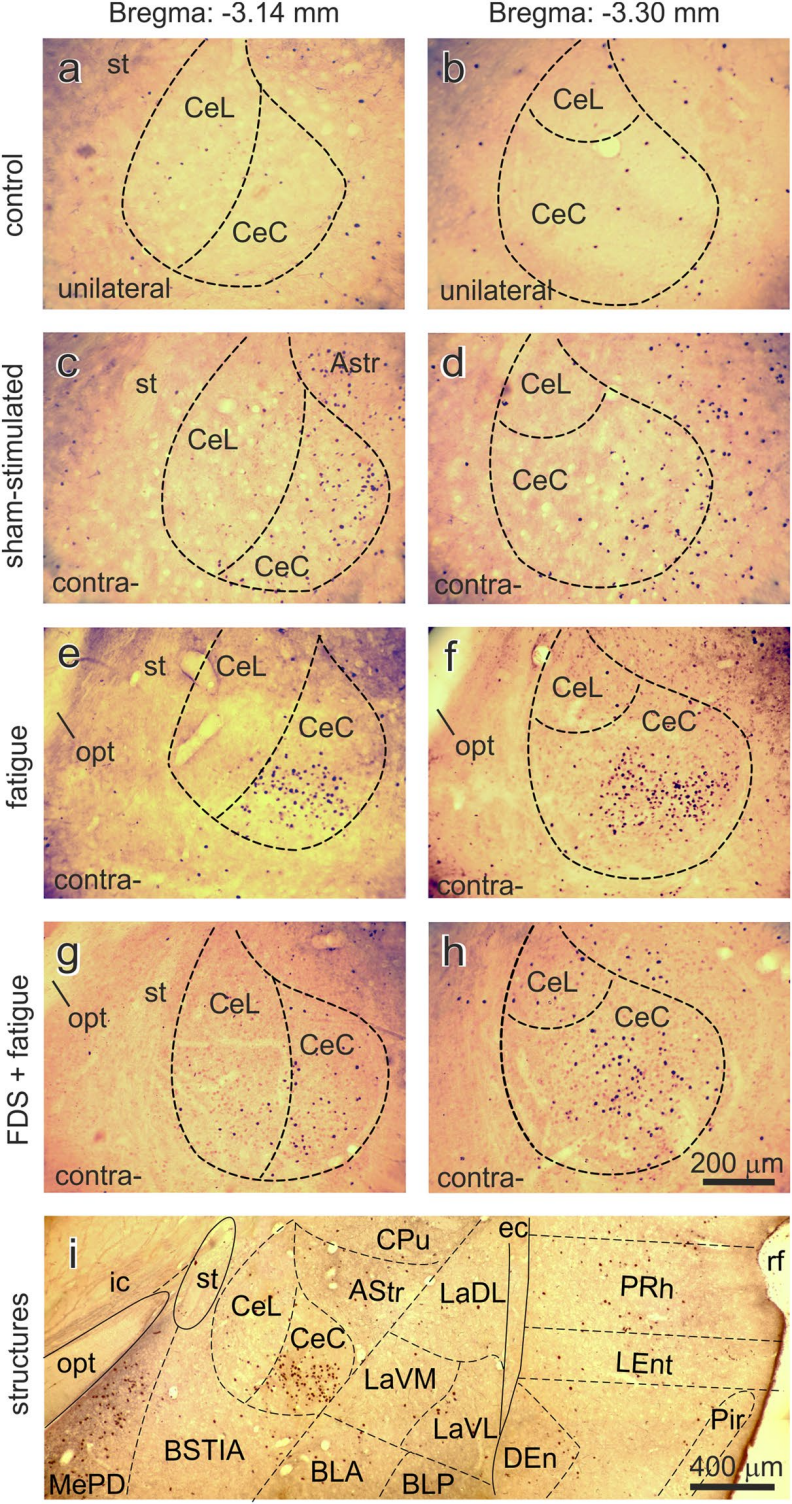
Photomicrographs of the Fos-immunohistochemically stained neurons within the central amygdaloid nucleus (contralaterally) at the levels −3.14 and −3.30 mm from the bregma, respectively: (**a**) and (**b**) – of the control rat; (**c**) and (**d**) – sham-stimulated animal (SS animal group); (**e**) and (**f**) – fatigue-induced rat (Fat2 animal group); and (**g**) and (**h**) – C_60_ fullerene pretreated and fatigue-induced rat (FP2 + Fat2 animal group). Example of Fat2 animal brain section at the levels −3.14 mm at low magnification is shown in (**i**). Photomicrograph for control animal group is presented unilaterally. Dark dots – nuclei of Fos-ir neurons. Structures according to the atlas^21^: amygdaloid nuclei (BLA – basolateral anterior, BLP – basolateral posterior, CeC – central capsular, CeL – central lateral, LaDL – lateral dorsolateral, LaVL – lateral ventrolateral, LaVM – lateral ventromedial, MePD – medial posterodorsal); AStr – amygdalostriatal transition area; BSTIA – bed nucleus of the stria terminalis, intraamygdaloid division; CPu – caudate putamen; DEn – dorsal endopiriform nucleus; ec – external capsule; ic – internal capsule; LEnt – lateral entorhinal cortex; opt – optic tract; Pir – piriform cortex; PRh – perirhinal cortex; rf – rhinal fissure; st – stria terminalis. Scale bars: 200 μm in (**h**) also appreciated for (**a–h**) fragments and 400 μm for (**i**).

In the animals in group 6, the number of positive neurons in the capsular part (CeC) and lateral division (CeL) of the CeA was approximately 5–8 cells per section at the different levels from the bregma (Fig. 8a,b). However, a notable accumulation of Fos-ir neurons was found in the contralateral capsular part (CeC) of the CeA at the levels −3.14 and −3.30 mm caudal to the bregma in the rats in the SS group. Thus, on these two sites, there were 24.2 ± 3.8 and 26.3 ± 2.3 Fos-ir neurons, respectively (Figs. 6b and 8c,d). In comparison with the sham-stimulated rats, the rats of the Fat2 group had a higher total number of Fos-positive neurons contralaterally in the CeC at both mentioned levels of the brain in response to the fatiguing muscle contractions (45.0 ± 3.7 and 55.7 ± 7.2 Fos-ir neurons, respectively, Figs. 6b and 8e,f,i). It should be noted that statistically significant differences in the mean number of activated neurons between the rats in the Fat2 and VP + Fat2 groups were not found, P > 0.05. In contrast, in the Fat2 rats with FDS, the preliminary injection of FDS demonstrated a contralateral decrease in the mean number of Fos-ir neurons in the CeC at −3.14 and −3.30 mm (Figs. 6b and 8g,h). Notably, contralateral prevalence in the mean number of Fos-ir neurons was detected within the CeA at both levels in the SS rats and animals which subjected electrical stimulation. Contralateral dominance in the mean number of labeled cells was also recorded in the lateral division (CeL) of the CeA in the animals in groups 9–12; however, the values of these groups did not significantly differ from those of the control group (Fig. 6b).

## Discussion

In this study, we investigate effect of FDS on changes in the level of neuronal activity within the lumbar spinal segments L4 and L5 segments and CeA under TS muscle fatigue development. In the electrophysiological part of the study, it was revealed that prior application of FDS induced notable changes in the TS muscle contraction force in the rats under fatigue development. It’s important to note that we used a low concentration of fullerene – 0.3 mg/kg. As previously was shown in various models *in vitro* and *in vivo*, C_60_ fullerenes and its derivatives can actively bind free radicals and display a powerful antioxidant properties of direct action^23,24^, and also modulate ion transport across membranes^25^. It is known, different C_60_ derivatives have different biological actions^26^. Nevertheless, important factors in the treatment with fullerenes and their derivatives are the size of the nanoparticles and their dosage. Kraemer *et al*.^27^ was found that C_60_ fullerenes size up to 450 nm (compared with particle size up to 200 nm) induced impaired spatial memory with a significant decrease in brain-derived neurotrophic factor protein levels and gene expression in rats after injection of the suspensions into the hippocampus. However, an enhanced antioxidant capacity was observed in both C_60_ treatments^27^. It was previously detected the maximum tolerated dose of C_60_ fullerene to be 5 g/kg for i.p. administration to rats^23^. Hydroxylated fullerene at a concentration of 100 mg/mL was also found to cause protein polyubiquitination in human umbilical vein endothelial cells, killed 58% of cells^28^, and exposure to a higher dose (137 mg/kg) resulted in embryo death in pregnant mice^29^. Zha *et al*.^30^ showed the effects of different concentrations of fullerene (fullerenol) on the CNS. It was revealed that low concentrations of this compound protect the hippocampal neurons in newborn rats from oxidative stress, while high concentrations of the fullerenol caused oxidant-induced apoptosis^30^.

In our investigation the dosage of 0.3 mg/kg FDS increased the active work duration of the muscle. Well-known that the enhancement of free radical processes is the main pathogenic factor in the development of skeletal muscle fatigue^31^ and that intensive physical activity induces overproduction of free radicals in muscle tissue^32^. At the same time, the use of exogenous antioxidants can lead to a significant decrease in skeletal muscle fatigue during prolonged and/or intense loads^33–36^. It was previously reported that the application of the C_60_ fullerenes can intensify anti-inflammatory effects^37^ and regulate the ATPase activity of actomyosin^38^. It has also been found that C_60_ fullerenes and their derivatives can enhance the protective functions of the body’s immune and antioxidant systems^39^. Apparently, application of the FDS may regulate the prooxidant-antioxidant balance in rat muscle tissue and effect on the dynamics of muscle contraction parameters.

The results of the immunohistochemical study demonstrate that in SS animals, the level of c-Fos expression was higher than that of the control animals. The insertion of the stimulating electrodes into the TS muscles in the sham-stimulated rats was followed by an increase in the basal level of c-Fos expression in the spinal segments and in the CeA. In comparison with the control or sham-stimulated animals, 30 min of fatiguing stimulation of the TS muscles induced distinctive changes in c-Fos expression patterns in ipsilateral laminae 1 and 5 of the lumbar (L4 and L5) segments and in the contralateral capsular part of the CeA in the Fat2 animals. As shown earlier, the amygdala receive nociceptive signals from the spinal cord neurons within lamina 1 and trigeminal nucleus caudalis via the spino-parabrachio-amygdaloid pain pathway, and provides nociceptive input to the CeA^40,41^. Via connections with the lateral and basolateral amygdaloid nuclei the CeA receives nociceptive inputs from thalamic and cortical areas^42–44^. Also known, that the amygdala can receive sensory and nociceptive inputs from the deep dorsal horn spinal neurons and/or neurons within lamina 10^18,45–49^. The CeA is a component of the descending pain-modulatory circuitry^8^. Efferent projections from the CeA are the main sources of information from the amygdala to the brainstem and hypothalamic structures that are involved in the descending modulation of nociception and generation of the autonomic and affective components of pain^50^. It has been shown that the CeA nucleus is involved in cannabinoid-induced antinociception in rats^8^ and that unilateral stimulation of the CeA, basolateral and medial amygdaloid in conscious rats results in a reduction in tonic formalin-induced pain^51^. The amygdala is rich in GABAergic neurons and GABA receptors, allowing the control of amygdala output through direct inhibition, feedforward inhibition and disinhibition^52,53^.

In our present study, it was shown that prior injections of FDS induced a notable decrease (by two or more times) in the mean number of Fos-ir neurons within ipsilateral laminae 1 and 5 in the studied segments and in the contralateral CeC compared with those of the fatigue-induced rats. It is known that muscle fatigue is accompanied by ionic changes in action potentials^54^ and various metabolic disturbances in skeletal muscles, such as the formation of reactive oxygen species^55^, the excessive production of lactic acid^56^, and lipid peroxidation^57^. However, C_60_ fullerenes, which are compounds with powerful antioxidant properties, can influence the prooxidant-antioxidant homeostasis of rat muscle tissue^5,6^.

Yamada *et al*.^26^ also investigated the effects of C_60_ fullerenes on the CNS in rats. In an *in vivo* study, C_60_ fullerenes were injected intraperitoneally or into the lateral brain ventricle. It was registered that the i.p. injection of the substance did not induce changes in the locomotor behavior of experimental animals, whereas intracerebral (i.c.) injection of C_60_ fullerenes increased such locomotor behavior and changed monoamine concentrations in the brain. It was registered decrease in the dopamine turnover rate in the hippocampus after i.p. application of C_60_ fullerenes and increase in the dopamine (in the hypothalamus, cerebral cortex and striatum) and serotonin (in the hypothalamus, cerebral cortex, striatum and hippocampus) turnover rate after i.c. fullerene injection. Thus, the different effects of i.p. and i.c. injections on the CNS allowed the authors to suggest that fullerene did not cross the blood-brain barrier^26^. In our case it does it mean that C_60_ fullerenes effect only like free radicals scavengers.

## Conclusion

It can be assumed that FDS decreases the concentration of free radicals in fatigued TS muscle tissue, which in turn reduces the transmission intensity of nociceptive information from the muscles to the spinal cord and amygdala. However, the influence of the descending pain-modulatory system is not excluded. Thus, prior injections of FDS can be one of the main factors affecting changes in the level of Fos immunoreactivity within the lumbar segments and the CeA.
